# Impact of redefining statistical significance on *P*-hacking and false positive rates: An agent-based model

**DOI:** 10.1371/journal.pone.0303262

**Published:** 2024-05-16

**Authors:** Ben G. Fitzpatrick, Dennis M. Gorman, Caitlin Trombatore

**Affiliations:** 1 Department of Mathematics, Loyola Marymount University, Los Angeles, California, United States of America; 2 Tempest Technologies, Los Angeles, California, United States of America; 3 Department of Epidemiology & Biostatistics, School of Public Health, Texas A&M University, College Station, Texas, United States of America; Leiden University: Universiteit Leiden, NETHERLANDS

## Abstract

In recent years, concern has grown about the inappropriate application and interpretation of *P* values, especially the use of *P*<0.05 to denote “statistical significance” and the practice of *P*-hacking to produce results below this threshold and selectively reporting these in publications. Such behavior is said to be a major contributor to the large number of false and non-reproducible discoveries found in academic journals. In response, it has been proposed that the threshold for statistical significance be changed from 0.05 to 0.005. The aim of the current study was to use an evolutionary agent-based model comprised of researchers who test hypotheses and strive to increase their publication rates in order to explore the impact of a 0.005 *P* value threshold on *P*-hacking and published false positive rates. Three scenarios were examined, one in which researchers tested a single hypothesis, one in which they tested multiple hypotheses using a *P*<0.05 threshold, and one in which they tested multiple hypotheses using a *P*<0.005 threshold. Effects sizes were varied across models and output assessed in terms of researcher effort, number of hypotheses tested and number of publications, and the published false positive rate. The results supported the view that a more stringent *P* value threshold can serve to reduce the rate of published false positive results. Researchers still engaged in *P*-hacking with the new threshold, but the effort they expended increased substantially and their overall productivity was reduced, resulting in a decline in the published false positive rate. Compared to other proposed interventions to improve the academic publishing system, changing the *P* value threshold has the advantage of being relatively easy to implement and could be monitored and enforced with minimal effort by journal editors and peer reviewers.

## Introduction

Concern with the inappropriate application and interpretation of *P* values has grown in recent years, especially regarding making multiple comparisons within one dataset and using *P*<0.05 to denote “statistically significance” differences [1–3]. Gelman and Loken observe that many datasets and analysis plans present the opportunity to make multiple comparisons without investigators purposefully and deliberately fishing for a *P* value below 0.05 [4]. However, the facts that most published results are positive and that an unusually large number of these barely pass the threshold *P*<0.05 have raised concern that many of these results may in fact arise from data dredging, also referred to as “*P*-hacking” [5–9]. Data-dredging denotes a deliberate and purposeful search for statistically significant results, with analysis continuing until at least one is found. This chance statistically significant result is then selectively reported in a manuscript as though it were the result of a prespecified test of a hypothesis.

A number of solutions to data dredging and selective outcome reporting have been proposed, including preregistration of study methods and analysis plans, data sharing, Registered Reports, blind data analysis, and adversarial collaboration. The latter two methods have not been widely adopted outside of psychology and physics, respectively [10,11]. While there is evidence that preregistration and data sharing can reduce the number of false positive results published by journals, these editorial practices have been undermined by a failure of many journals to enforce preregistration and data sharing policies and by some study registries allowing retrospective registration [12–14]. As for Registered Reports, interpretation of study findings indicating that it produces more results supporting the null is difficult as it cannot be ruled out that researchers especially concerned with selective outcome reporting bias and/or inclined to test “risky” hypotheses are more likely to use this format [15,16].

A formal proposal that the threshold for “statistical significance” be changed from *P*<0.05 to *P*<0.005 was made by Benjamin and other statisticians and research methodologists in 2018 [17]. It is premised on the idea that “statistical standards of evidence for claiming new discoveries in many fields of science are simply too low” [17, p. 6]. The practice of associating statistically significant findings with *P*< 0.05 will result in a high false positive rate, they argue, irrespective of other problems in study design, data analysis, and reporting of results. In support of their argument, Benjamin et al. state that a two-sided *P* value of 0.05 corresponds to Bayes factors in favor of the alternative hypothesis in the range of 2.5 to 3.4, which is “weak” or “very weak” evidence. In contrast, a two-sided *P* value of 0.005 is in the “strong” to “substantial” range of Bayes factor recommendations. They also present a table showing the association between the two *P* value thresholds, study power, prior odds of the alternative hypothesis, and the false positive rate. For example, with prior odds of 1:10 and power of 1.0, the false positive rate drops from 33% with a *P* value threshold of 0.05, over a range of values of statistical power, to 5% with a threshold of 0.005. Lakens et al. [18] question the underlying assumptions and data used in arriving at each of these justifications (e.g., the prior odds estimate used in calculating the false positive rates for each alpha level is based on data from just 73 studies from the *Reproducibility Project*: *Psychology*).

Opinions as to the effect of changing the *P*-value threshold on data dredging and *P*-hacking vary. Ioannidis [1] considers it to be a “temporizing measure” to dam the flood of positive results, but likely to be more beneficial than harmful. On the other hand, Amrhein and Greenland [19] contend that the new threshold will lead to “more intense *P* hacking and selective reporting”. Benjamin et al. [17] acknowledge that an investigator can still engage in such analytic practices while using a *P*<0.005 threshold but contend that the likelihood of a “statistically significant” chance finding emerging from these analyses is lower than with a 0.05 threshold. Moreover, it is likely that the longer the data dredging exercise continues, the more easily identifiable it will be to those reading a publication in which such results are reported; that is, investigators will be forced into conducting more extreme analyses as the search for a statistically significant result continues (e.g., removing study groups or assessment points from the analysis, even when the study is registered) [e.g., 20,21].

A handful of studies have assessed the effects of the proposed change in threshold on the number of statistically significant results reported using *P* values from already published studies. Based on a large text-mining study of many thousands of *P* values published over 25 years, Ioannidis estimated that changing the *P* value threshold from 0.05 to 0.005 would remove about one-third of the statistically significant results of past biomedical literature [22,23].

In a series of studies, Vassar and colleagues examined the impact of changing the threshold on randomized controlled trials (RCTs) published in general medical, orthopaedic trauma, and orthopaedic sports medicine journals [24–26]. The results of these studies are summarized in Table 1, along with a study by Thakur and Jha [27] that examined changing the *P* value threshold on results from 123 RCTs pertaining to chronic rhinosinusitis and a study by Khan et al. [28] that focused on 72 RCTS from high impact general medical and cardiology journals. Across the five studies, the range of *P* values that retained statistical significance with a 0.005 threshold was 38.9–70.7%.

**Table 1:** Summary of results from studies that assessed changing the P-value threshold.

Study	Studies Included in the Analysis	Date Studies Reviewed were Published	Primary Endpoints with a *P* value	Statistically Significant at *P*<0.05 (%^a^)	Remained Statistically Significant at *P*<0.005 (%^b^)
Wayant et al. [26]	203 Phase 3 RCTs published in JAMA, *Lancet*, and *New England Journal of Medicine*	01/01/17-12/31/17	272	174 (64%)	123 (70.7)
Johnson et al. [25]	48 RCTs in *Journal of Orthopaedic Trauma*, *Injury*, and *Archives of Orthopaedic & Trauma Surgery*	1/01/16-01/31/18	124	49 (39.5)	25 (51.0)
Evans et al. [24]	132 RCTs in *American Journal of Sports Medicine*, *Arthroscopy*, and *Knee Surgery*, *Sports Traumatology*, *Arthroscopy*	1/01/16-12/31/17	275	126 (45.8)	49 (38.9)
Khan et al. [28]	72 RCTs in *Circulation*, *Circulation*: *Heart Failure*, *European Heart Journal*, *European Journal of Heart Failure*, *JACC*: *Heart Failure*, *JAMA*, *Journal of the American College of Cardiology*, *Lancet*, *New England Journal of* Medicine	1/01/13-12/31/17	90	37 (41.1)	20 (54.1%)
Thakur and Jha [27]	123 chronic rhinosinusitis RCTs identified in PubMed	01/01/11-12/31/20	168	80 (47.6)	43 (53.8)

^a^ % of total primary endpoints reported in the papers reviewed with a *P* value that were statistically significant at *P*<0.05.

^b^ % of *P* values that were significant at *P*<0.05 that remained statistically significant at *P* < .005.

The studies in Table 1 were focused on recent RCTs published in top medical journals and a majority would have been registered. Such studies are most appropriate for null hypothesis statistical testing using *P* values. In contrast, research focused on data dredging, *P*-hacking and the clustering of *P* values just below 0.05 has typically examined studies in psychology, biology and political science that are unlikely to be preregistered and many of which will use study designs other than RCTs [e.g., 7,9,29,30]. Benjamin et al. [17] recommend that research using non-experimental designs, especially exploratory research that tests numerous hypotheses, should employ even lower *P* value thresholds than 0.005. It is unknown what percent of *P* values from non-experimental studies that were statistically significant with the traditional 0.05 threshold would remain so with a 0.005 threshold, let alone one more stringent.

Assessing the potential effects of changes in *P* value thresholds on the validity of published results is difficult as traditional empirical methods involving experimental manipulation are not feasible. Such constraints apply to studying many aspects of the current academic incentive system and proposed changes to realign this in ways that produce higher quality research and more valid published findings. This has led to the application of simulation models to estimate the effects of the “publish or perish” academic culture on the quality of published scientific research and the potential of proposed changes to the publication process to improve this. An influential work in this area is that of Smaldino and McElrealth [31] which describes a competitive agent-based model (ABM) [32] comprised of research laboratories that survive based on their ability to publish a high volume of papers while expending low effort, albeit many of which report false positive results. The model demonstrates that this way of conducting research, called “bad science”, can rapidly spread through a group of laboratories, and become its dominant approach. The main results of the model were recently replicated [33], and its framework has been used to conduct virtual experiments of interventions such as auditing of research facilities, making publication of negative results more prestigious, improving peer review, assigning research funds randomly or according to methodological integrity, and researchers expending effort on the selection of strong hypotheses through theory development [34–36].

The aim of the current study is to examine the statistical process of empirical science using an ABM, building on the work of Smaldino and McElrealth [31]. Here the agents are researchers who test hypotheses and publish positive findings. With this model we explore the impact of *P*-hacking and modified *P* value thresholds on false positive rates in the literature.

We should be clear as to two aspects of what it is we are modeling. First, the disciplines we have in mind are those in which a culture of “you can publish if you found a significant effect ‘‘ prevails, thereby encouraging multiple statistical testing (i.e., data dredging or *P*-hacking) to obtain such effects [37]. Disciplines in which such concerns have been raised include biomedical sciences, ecology, psychology, and biology [37–40]. The percent of published positive results in such “soft” disciplines is estimated to be over ninety, especially in applied areas of research [41]. Depending on study design, sample size, specificity of hypotheses, analytic flexibility, and other methodological features, most of these disciplines’ published positive results may be false [37,42–44]. While even the hardest of sciences are not immune to confirmation bias, multiple testing using *P* values to determine “significance” is not among the questionable research practices used to achieve desirable results, and therefore the solutions in these disciplines are different, albeit with potential application to the soft sciences [45,46].

The second clarification involves the type of null hypothesis significance testing (NHST) we model. There is a large literature detailing how NHST as reported in most published papers produced by researchers in the disciplines described above is a problematic amalgam of Fisher’s significance test and the hypothesis testing approach described by Neyman and Pearson [47–49]. The problems with this hybrid have been clearly explained but are not of immediate concern to us as we are presenting an idealized model of what researchers in disciplines such as biomedical science and psychology *typically do* when hypothesis testing rather than what they *should do*. Accordingly, in the current model the agents structure their studies as a null hypothesis statistical test, and they reject the null hypothesis on the basis of a *P* value of 0.05 or below.

## Methods

Building on the model of Smaldino and McElreath [31], we populate our ABM with *N* researchers that dynamically perform experiments to generate new results. Each researcher is characterized by a vector of state variables: effort, number of hypotheses per experiment, value, age, and effect size. The first two values denote the researcher’s methods, in the sense that the amount of effort and the number of “bites at the apple” (i.e., attempts to find a statistically significant result) comprise the variables that drive publication outcomes, as will be seen below.

### Effort

**Fig 1:**
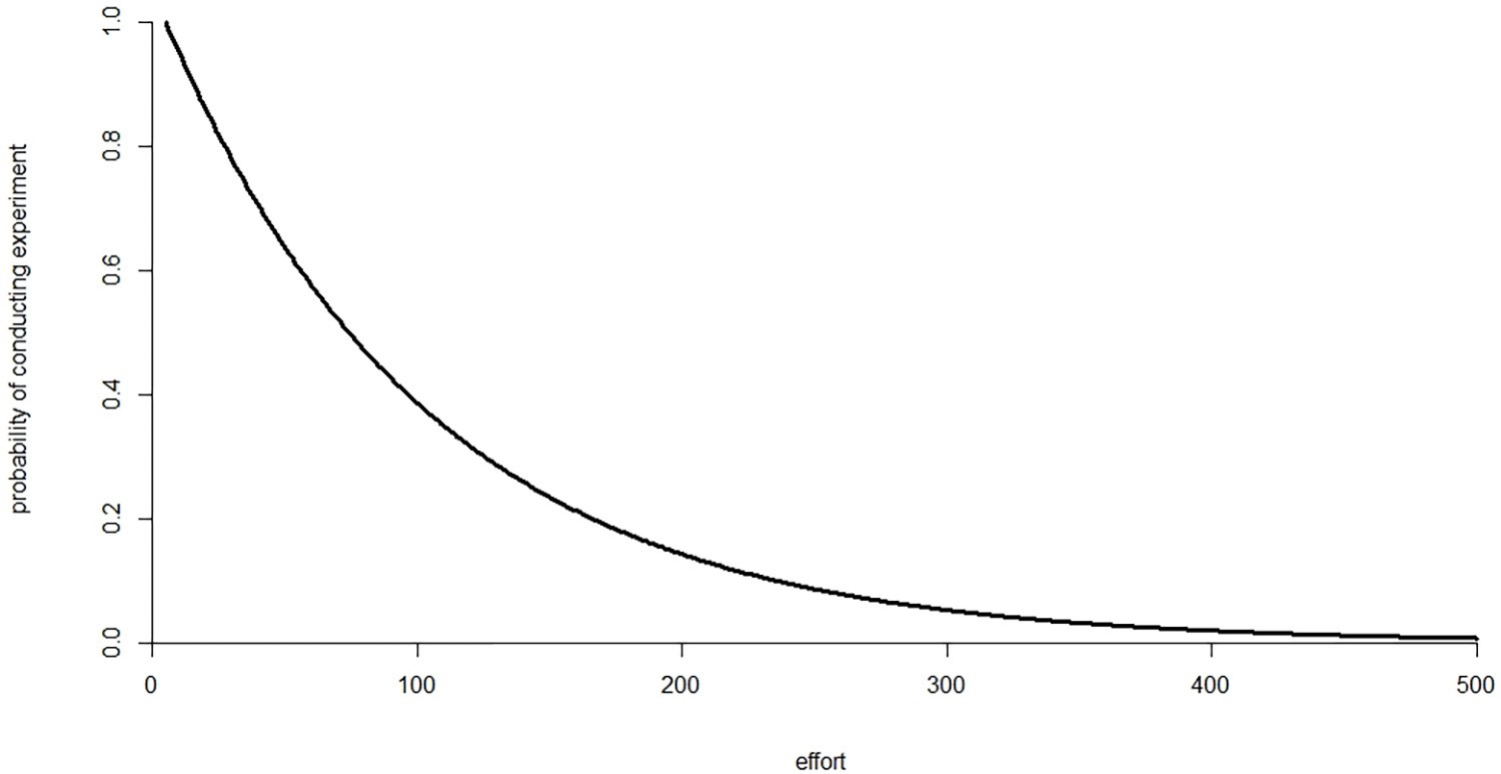
Functional relationship between agent effort and probability of conducting an experiment.

In our model, effort impacts the research process in two ways: the time it takes to perform an experiment and the power a study can achieve. These two impacts are in tension–more effort means potentially slowed productivity but improved true positive results. Smaldino and McElreath [31] model the probability of conducting a study during a time step with a convex, decreasing function. Our functional form is different but maintains the convex, decreasing shape:

$$p=exp(-\eta [E-E_{min}]),$$

in which *E* denotes effort, *E*_*min*_ denotes the lower bound on effort, and *η* is a tunable model parameter. Greater effort means smaller probability of false positive results. Fig 1 illustrates this functional relationship.

### Number of hypotheses

It is in this component that our model is quite different from that of Smaldino and McElreath [31]. We simulate a statistical hypothesis test, a modeling choice that allows us to investigate the reduced *P* value threshold intervention. The number of statistical hypothesis tests performed models the process of *P*-hacking, repeatedly testing in hopes of finding something to be statistically significant.

### Value

If the researcher performs a hypothesis test that achieves a sufficiently small *P* value, then that positive finding results in a publication. The researchers’ value is the total number of publications over the course of their careers.

### Age

The number of time steps in the simulation that the researchers are active is their age.

Time progresses in discrete fixed steps, during which each researcher:

- applies an amount of effort in order to conduct an experiment (or not);
- collects data to analyze;
- tests one or more hypotheses;
- publishes any positive finding;
- checks the publication rates of other researchers to identify “better” methods.

In addition, researchers “quit” or “retire” at random with an exponential rate, and new researchers enter the workforce to maintain a fixed population size of *N* researchers.

### Effect size and hypothesis testing in the model

In order to study the impact of the *P* value threshold change, we must simulate the process of statistical hypothesis testing, which involves two types of error: type I or false positive and type II or false negative. In aggregate, *P* value thresholds define the acceptable probability of false positive findings.

An important aspect of the simulation is the modeling of “truth.” To simulate false positive rates within a hypothesis testing context, we must select a means of generating “true” and “false.” In this model, each researcher conducts a number of independent-sample *t*-tests, the simplest test comparing a control group to an experimental or treatment group. To simulate these *t*-tests, we generate true Cohen’s *d* effect sizes from an exponential distribution:

$$P[d\leq x]=1-exp(-\frac{x}{d_{0}})$$

in which *d*_0_ is a tunable modeling parameter. Smaller effect sizes are more likely, and larger effect sizes are less likely in this model.

To generate experimental truth, we model an effect size greater than *d*_*min*_ (a tunable parameter) to yield an experiment that is a true positive. Within this modeling structure, the prior probability of the null hypothesis being true is

$$P[H_{0}]=1-exp(-\frac{d_{min}}{d_{0}}).$$.

**Fig 2:**
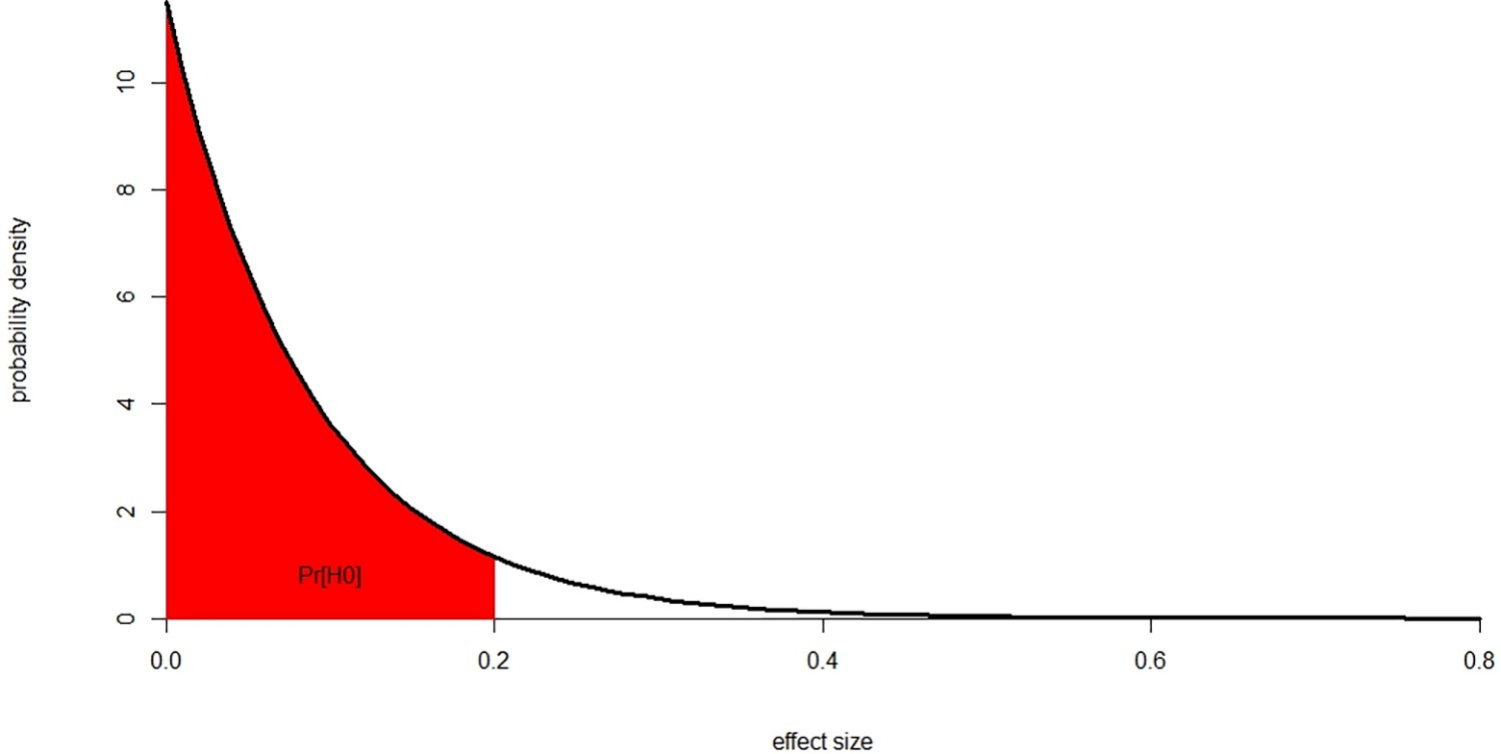
Probability density used to generate effect sizes.

For example, if we set *d*_*min*_ = 0.2, which is a small effect according to Cohen [50], and we set 80% as the prior probability of the null, we have *d*_*0*_ = -0.2/ln(0.8) = 0.1243. In general, the exponential scale parameter is determined from the minimum effect size and the prior probability by the formula $d_{0}=-\frac{d_{min}}{\ln\left(1-P[H_{0}]\right)}.$ Fig 2 illustrates the probability density we use to generate effect sizes in the model.

This model treats *d*_*min*_ as a minimally relevant effect size, which is the smallest effect that would “justify associated costs, risks, and other harms” [51, p. 251]. An experiment simulated with an effect size below *d*_*min*_ would be considered as arising from a true null hypothesis; likewise, an experiment simulated with an effect above *d*_*min*_ would be a false null. Once the experiment is simulated, and data are obtained, the *t*-test is performed. A true positive, then, will happen when the point-null hypothesis of zero effect is rejected, in an experiment simulated with an underlying effect size above *d*_*min*._ A false positive will come about when the null is rejected in a simulation with an underlying effect at or below *d*_*min*._

Of course, this definition of truth differs from the point-null that the effect size is 0 (versus the alternative that it is not). An alternative modeling choice would be to simulate truth of 0 effect sizes with some *a priori* probability and non-zero effect sizes with the complementary probability. But as Gelman and Carlin [52, p.900] remark, “[r]ealistically, all statistical hypotheses are false: effects are not exactly zero, groups are not exactly identical, distributions are not really normal, measurements are not quite unbiased, and so on.” Toward that end, we choose to model truth via minimally relevant effect sizes.

Within this structure there are many ways to simulate effect sizes for researchers. In our model, we simulate a single *d* value for each researcher, say *d*_*i*_, *i* = 1,2,…,*N*. This value serves as a scale parameter for simulating effect sizes for individual experiments. That is, at each time step, Researcher *i* will obtain $d_{i1,}d_{i2,}\ldots ,d_{ik_{i}}$, effect sizes for the multiple hypotheses 1,2, …, *k*_*i*_. Researcher *i* then obtains *k*_*i*_ t-statistic values randomly sampled from the noncentral t distribution with 2*E_*i*_-2 degrees of freedom and noncentrality parameters

$$\lambda_{ij}=d_{ij}\sqrt{E_{i}}/2.$$

At this point in the simulation, each researcher has obtained a number of *T* values. A *P*-hacking researcher would ask if at least one of these is greater than the critical *t*-value for the null hypothesis at the threshold level *α*. If so, the researcher would claim a positive outcome and publish. To determine whether or not that result is a true positive in the simulation, we check that (1) the effect size was at least *d*_min_ and (2) that the *T* value exceeded a *P* value-corrected critical *t*-value using the Šidák correction [53].

$$\alpha_{S}=1-({1-\alpha )}^{1/k}$$

in which *k* denotes the number of hypotheses (or *T* values obtained by the researcher). If these two conditions are met, the result is a true positive for the purposes of simulation. Otherwise, the result is a false positive. Any positive finding increments the researcher’s value by one unit. Negative results do not count towards the researcher’s value.

To be specific, *P* values computed herein correspond to the divergence *P* value as defined in Greenland [48]. That is, *P* values are computed as probability of future, replicated test statistics exceeding the value of the test statistic computed for the data at hand, under the condition that the null hypothesis is true. To the best of our knowledge, most statistical software packages that provide *P* values compute them in this manner.

The parameters of the model are given in Table 2.

**Table 2:** Model parameters.

Parameter	Description	Default Value
N	number of researchers	2000
e0	initial effort (applied by all researchers)	20
k0	initial number of hypotheses	1
emin	minimum effort	5
kmin	minimum number of hypotheses	1
emax	maximum effort	500
kmax	maximum number of hypotheses	50
σ_E_	standard deviation for effort evolution	10
σ_k_	standard deviation for number of hypotheses evolution	2
P[H0]	prior probability of a true null	0.8
dmin	mimimally relevant effect size (smallest effect for false null)	0.2
η	influence of effort on experiment rate	0.01
pRetire	rate at which researchers retire	0.002
LM	additional fraction of top researchers for evolutionary offspring	0.1

## Results

**Fig 3:**
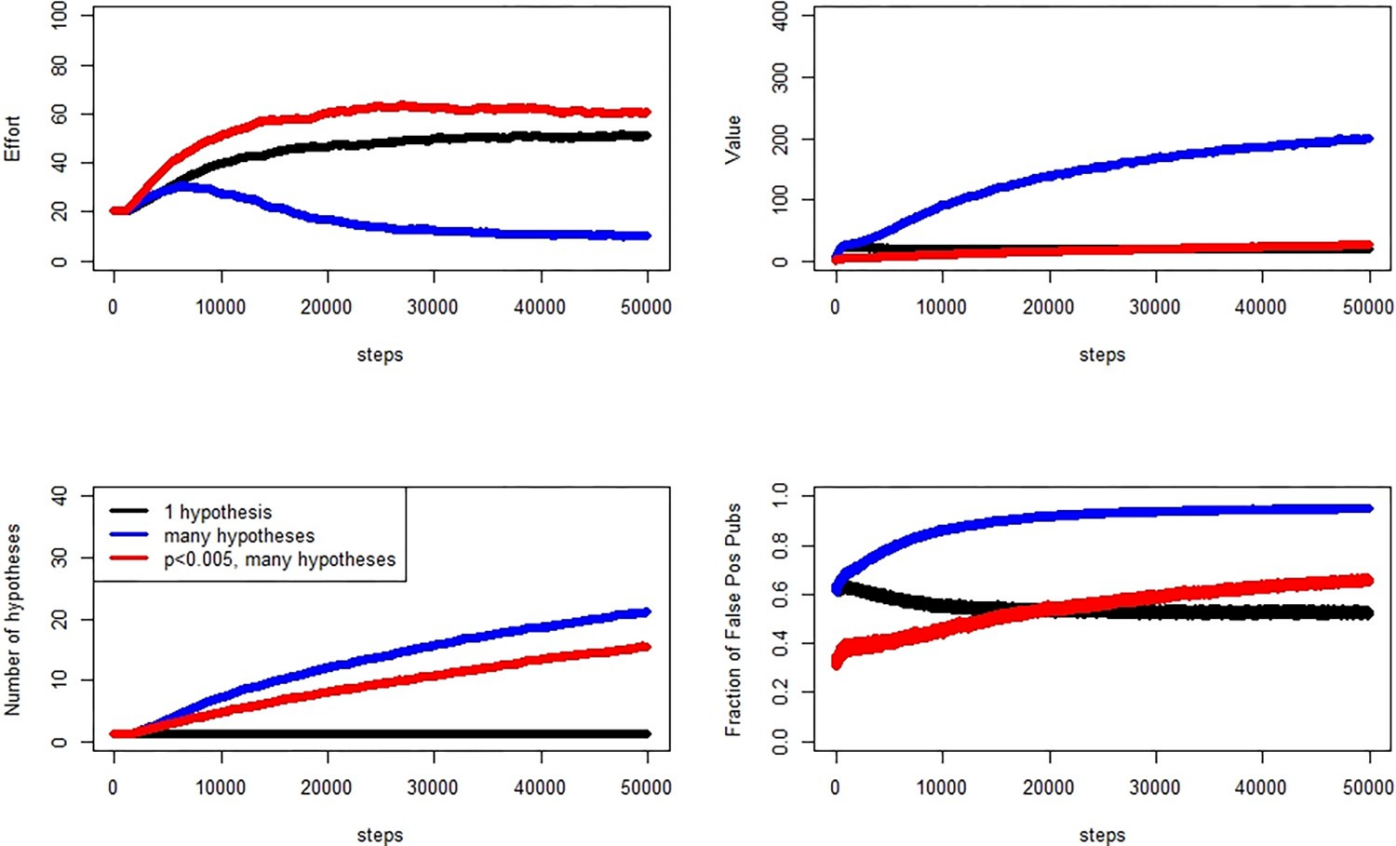
Evolution of effort, number of hypotheses tested, false positive publication rate, and value (clockwise from top left), Pr[H0] = 0.9. Quantities are medians across 2000 researchers, averaged over 100 simulation replicates.

We present three sets of model runs, organized by the prior probability of the null being 0.9, 0.8, or 0.5. In each set of runs, we compare three different scenarios. The first is an “ideal” scenario, in which each researcher tests exactly one hypothesis with each experiment. If that experiment’s analysis results in a *P* value of less than 0.05, the researcher publishes. In the simulation, we know which hypotheses are actually true, so we tabulate the fraction of published works that are false positives. In the second, researchers may test multiple hypotheses, and if at least one of these results in a *P* value below 0.05, that experiment is published. In this scenario, researchers are not using any type of *P* value correction for multiple testing. However, in the simulation, true positives are determined using the Sidak correction: “truth” is simulated, but “positive” for the purpose of “true positive” is computed with the Sidak correction. The third scenario follows the second except that we apply the intervention of reducing the P value threshold to 0.005. Fig 3 illustrates the evolution over 50000 time steps and 100 evolutionary replicates. Panels in the figure show the medians across researchers and replicates at each time step.

In this set of simulations, we use Pr[H0] = 0.9, wherein the majority of simulated experiments will come from effect sizes below the threshold for “truth.” We see that P-hacking continues at the 0.005 threshold, but that the false publication rate declines from the 0.05 threshold false publication rate.

**Fig 4:**
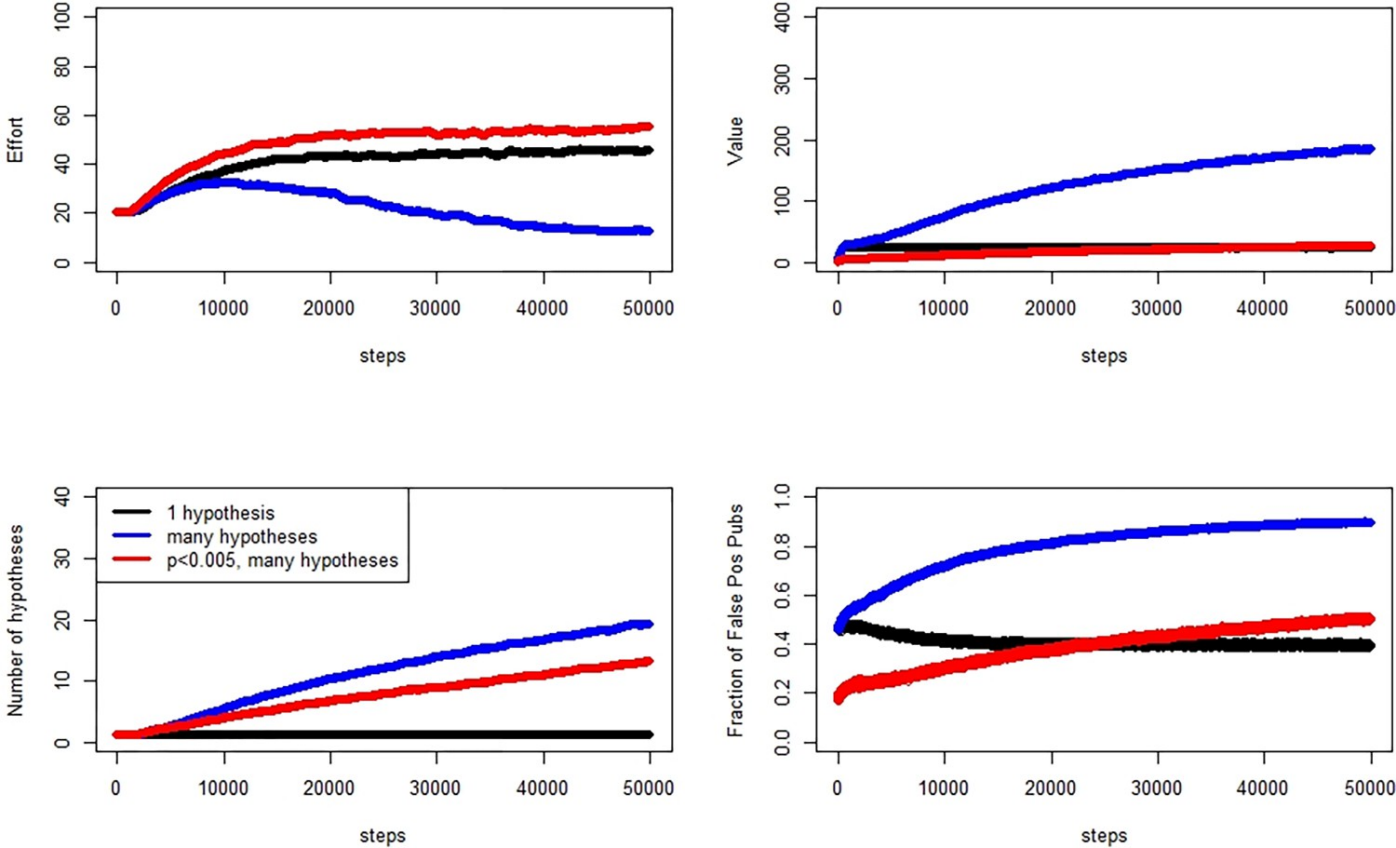
Evolution of effort, number of hypotheses tested, false positive publication rate, and value (clockwise from top left), Pr[H0] = 0.8. Quantities are medians across 2000 researchers, averaged over 100 simulation replicates.

**Fig 5:**
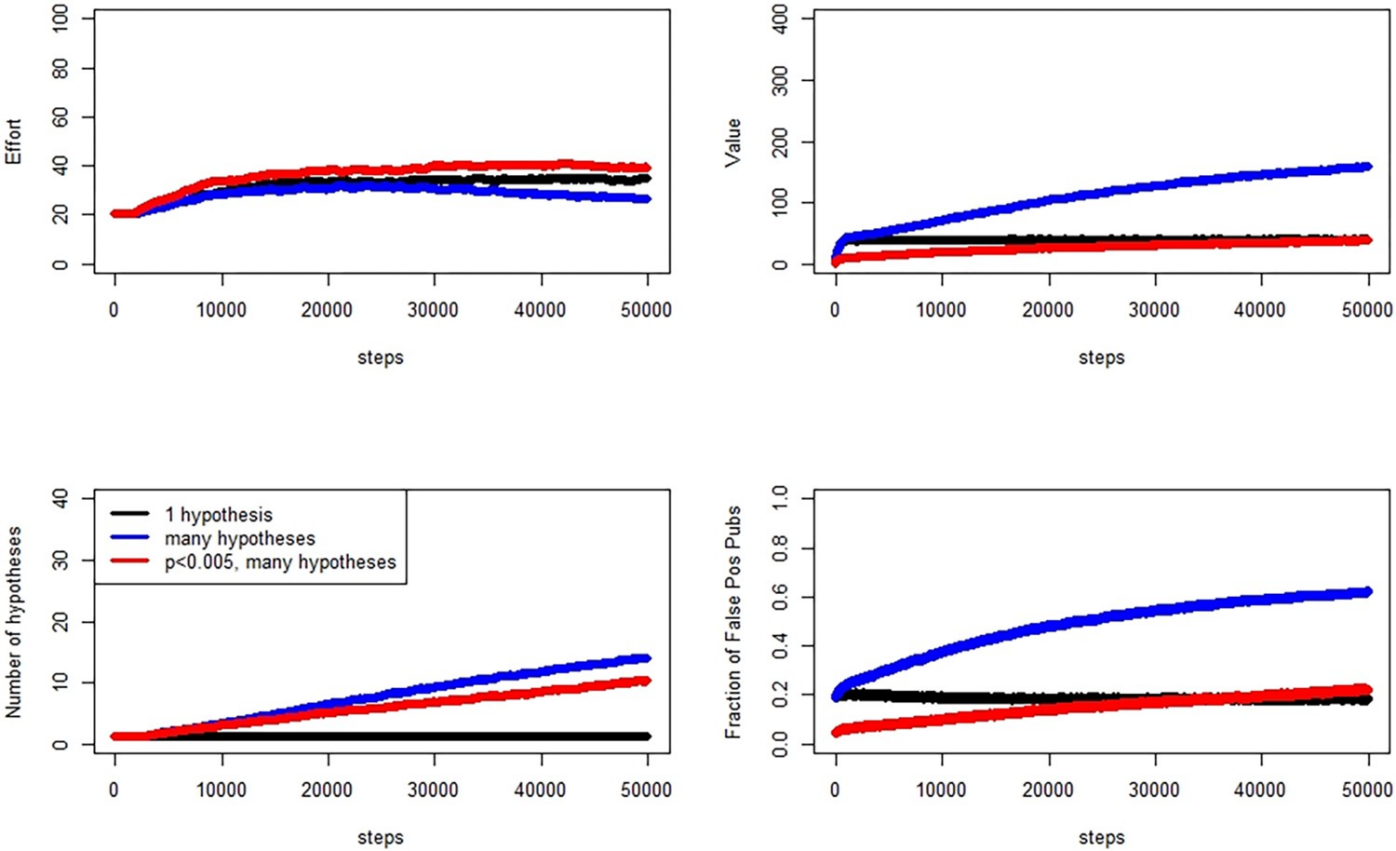
Evolution of effort, number of hypotheses tested, false positive publication rate, and value (clockwise from top left), Pr[H0] = 0.5. Quantities are medians across 2000 researchers, averaged over 100 simulation replicates.

In a second simulation, presented in Fig 4, we examine the prior probabilitiy of the null set to 0.8, and in Fig 5 we show results for the prior probabilitiy of the null set to 0.5.

**Fig 6:**
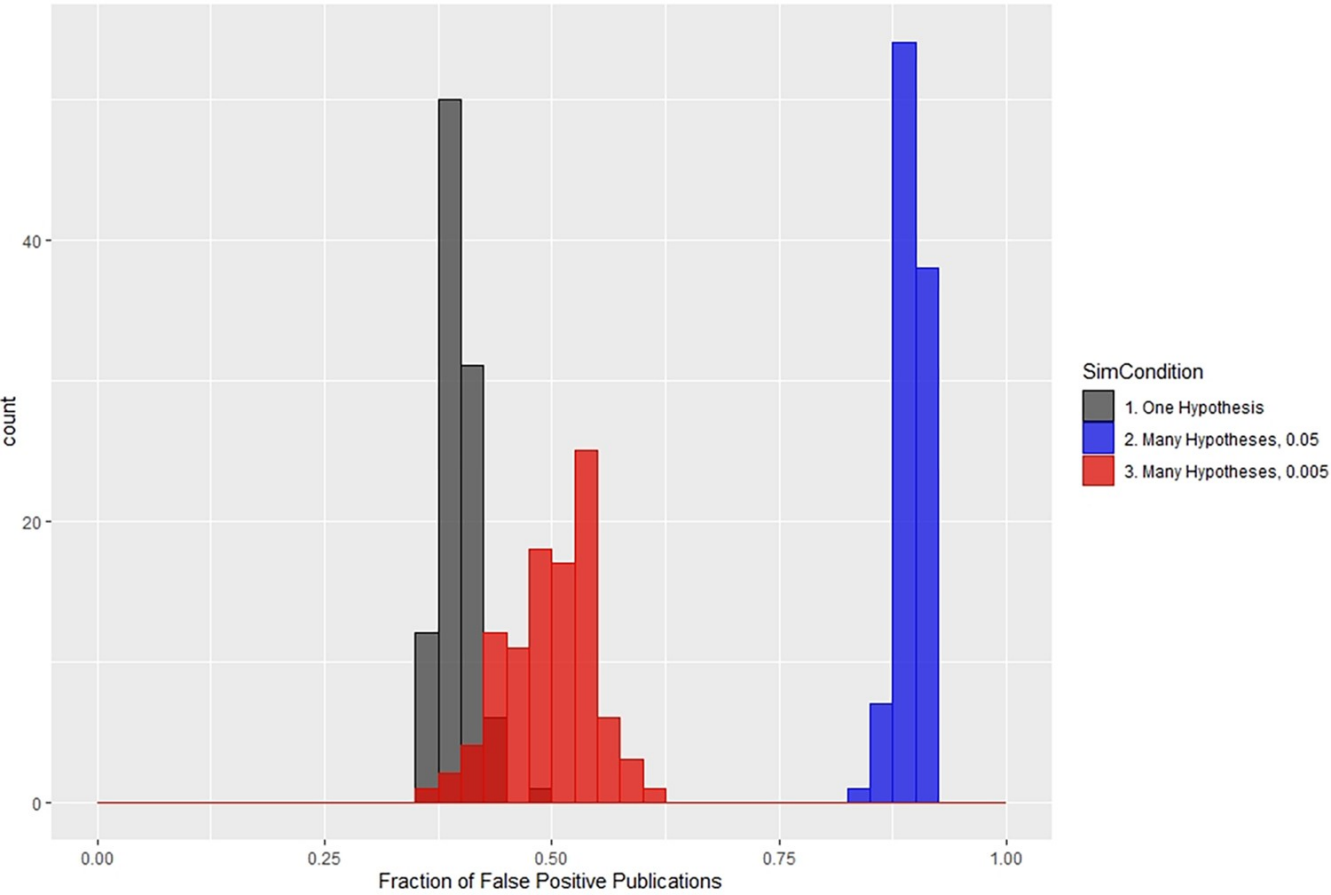
Distribution of false positive publication rates for the three scenarios.

In Figs 3–5, medians are graphed over time, but variation around the median is also of interest. To illustrate the variation in these quantities, we follow up with some histograms for the case of Pr[H0] = 0.8. In Fig 6 we show the system’s false positive publication rate at the final time step, as a histogram over 100 simulation replicates. For each replicate, we compute the cumulative number of false positives over the current researchers, and we divide by the cumulative number of publications (that is, true positives plus false positives) of the current researchers. We generate a histogram for each of the three simulation scenarios. For the single hypothesis scenario, values cluster in the center of the distribution, with a maximum frequency occurring near a false positive rate of 0.52. The distribution under the 0.005 scenario bears a resemblance to this, with a mode near 0.54 and somewhat more variation. In contrast, the 0.05 distribution clusters around 0.875–0.9, indicating a greater number of false positive results among the researchers at the final step of the model.

**Fig 7:**
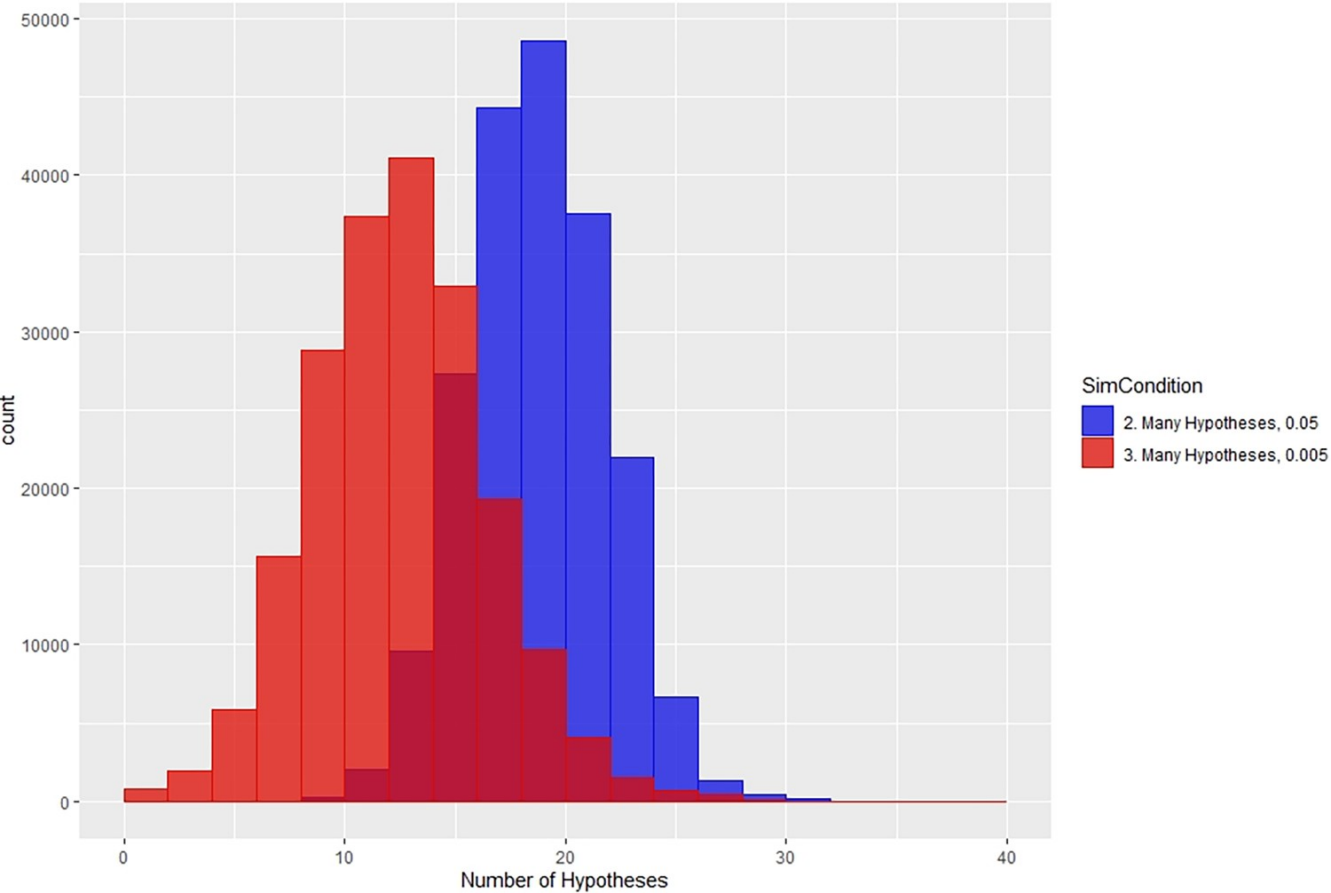
Distribution of number of hypotheses for the three scenarios (insert shows only the 0.05 and 0.005 threshold).

Variation in the number of hypotheses tested for each of the three scenarios is shown in Fig 7. The number of hypotheses for each researcher is tabulated over the 100 replicates and the 2000 researchers. This shows the continuation of testing multiple hypotheses, that is *P*-hacking, under the 0.005 scenario. For both 0.05 and 0.005 thresholds, the distributions show a bimodal shape with one peak at the low end and a spread of values that skew slightly toward larger values. In both cases, the number of attempted hypotheses tapers off for values larger than 15.

**Fig 8:**
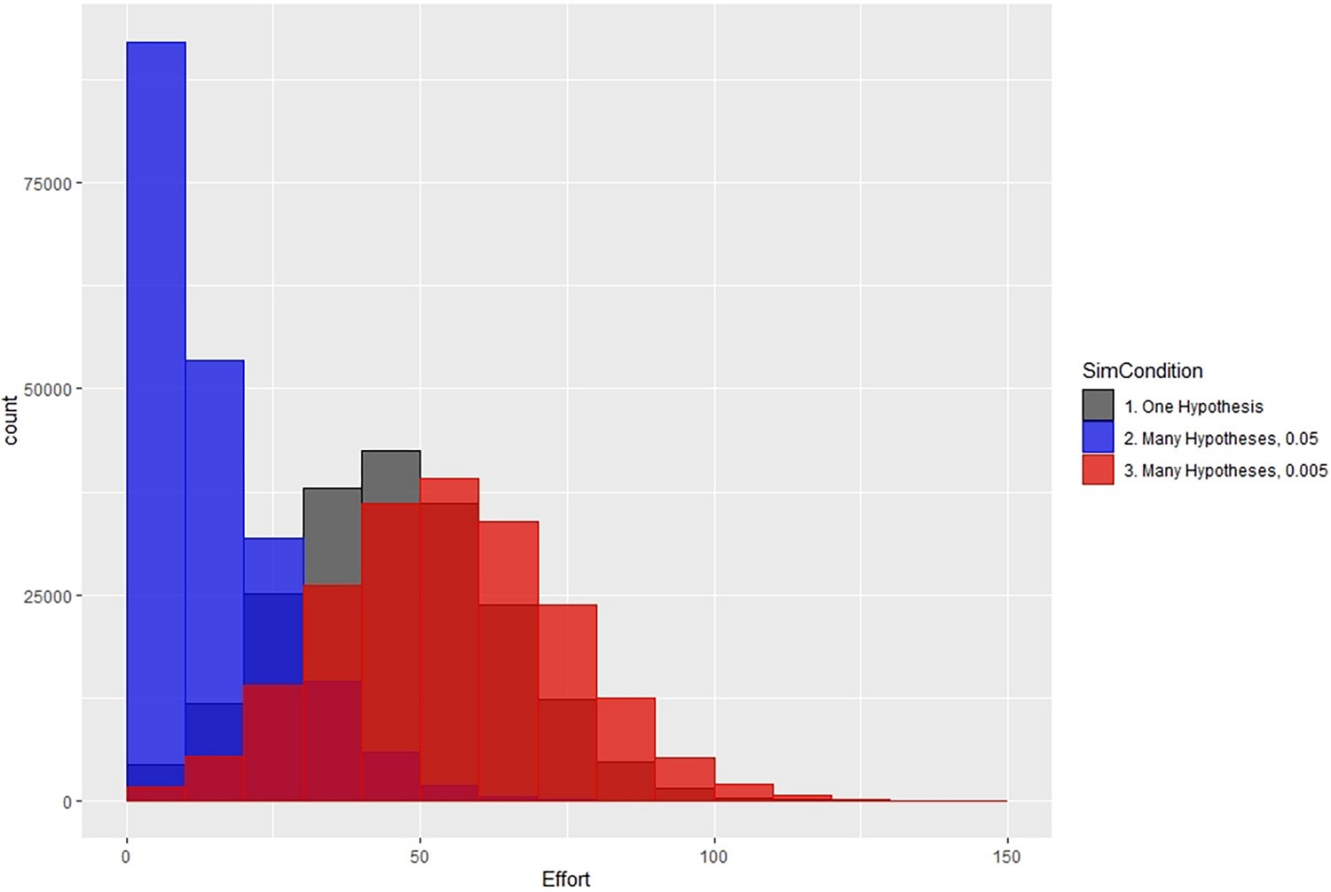
Distribution of effort for the three scenarios.

Variation in the amount of effort for each scenario is shown in Fig 8. The effort for each researcher is tabulated over the 100 replicates and the 2000 researchers. The distribution of 0.05 multiple hypotheses tested with a threshold of 0.05 resembles that of the single hypothese, but with higher peaks. Changing the threshold to 0.005 produces a more even distribution of effort with lower peaks than the 0.05 threshold.

## Conclusions

The results of the simulations generally support Ioannidis’ [1] view that a more stringent *P* value threshold can serve to dam the flood of published positive results. While, as Benjamin et al. [17] acknowledge, investigators can still engage in *P*-hacking, the false positive rate declines using the 0.005 threshold while the effort expended by investigators increases substantially. Overall, their productivity is reduced, but there is an improvement in production of true positive results.

While the results of our simulation do suggest that reducing the *P* value threshold may reduce the number of false-positive publications, we must note that this model has a number of limitations. First and foremost, it serves as an idealized version of the statistical aspects of the research process. Simulated researchers herein are applying a simple (two-sided) point-hypothesis test that is an appropriate test for the simulated data. *P*-hacking is modeled as a simple repeated generation of data for that test. There is no “garden of forking paths” that might lead researchers to search for a statistical method based on the observed data [4]. Moreover, researchers are not permitted to remove or add data in order to obtain positive results [54]. Certainly these (and other) unmodeled researcher activities could serve to moderate the effectiveness of the 0.005 intervention.

As noted in several recent papers, academic publishing can be viewed as a production system which currently incentivizes engagement in problematic behaviors such as data dredging, *P*-hacking, HARKing, and selective outcome reporting [55–58]. Using such a systems framework allows for the identification of leverage points which can be the target of interventions designed to improve research quality and integrity [59]. Such leverage points in the system will vary in the extent to which they are amenable to change and the magnitude and type of improvements in outputs that can be expected should such change successfully occur.

Preventive interventions targeted at leverage points can have a positive effect by reducing the extent to which individuals within the system engage in an unwanted behavior and/or by reducing the negative consequences (or harms) of engaging in the behavior. The simulations presented here indicate that the introduction by academic journals of a more stringent *P* value threshold for “statistical significance” has the latter effect: researchers continue to *P*-hack but the number of false discoveries that enter the published literature as a result of this practice is reduced by more than a half compared to using a 0.05 threshold when effect sizes were randomly generated.

One thing to consider when deciding if the reductions in false positive results observed in the simulations is worth pursuing is that a change in the *P* value threshold is a relatively simple intervention to introduce into the academic publishing system and one that could be monitored and enforced with minimal effort by journal editors and peer reviewers. Essentially, it involves changing one arbitrary threshold of “statistical significance” for another, albeit a less familiar one. In practice, it would require journals announcing in their instructions to authors that *P*<0.005 now constitutes statistical significance, running each submitted manuscript through a computer program to ensure the new threshold was adhered to (and returning to authors those that did not), and requesting that peer reviewers also ensure the 0.005 significance threshold was used in the analyses reported. In short, changing the *P* value threshold has the appeal of being an intervention with a clear target that involves an easy behavioral change and, if implemented widely, can reduce the false positive rate, albeit not entirely eradicating *P*-hacking.

While other interventions designed to improve research integrity and quality have greater potential impact, the feasibility of their widespread implementation, adherence, and enforcement is questionable. For example, in an ideal world every genuine *a priori* hypothesis-testing study would be written-up in the form of a Registered Report. This essentially embeds pre-registration in the publication pipeline and eliminates the incentive for researchers to data dredge when writing-up the results of their studies [15,16]. This format appears successful in reducing the publication of positive results and, consequently, reducing the rate of false discoveries [60,61]. However, Registered Reports have not enjoyed widespread adoption among journals: in early 2022, Chambers and Tzavella [15] reported that only 300 journals offered this as a publishing option, with just 94 of these having published a total of 591 final manuscripts reporting study results.

Prospective registration is another proposed intervention that, in principle, can greatly reduce *P*-hacking and selective publication of positive results [62,63]. While this has been more widely adopted by academic journals than the Registered Reports format, adherence by authors and enforcement by journals is suboptimal and some registries allow retrospective registration and alterations of protocols after a study is underway or even complete [13,14].

To the extent that it reduces the number of false positive results that find their way into the published literature, changing the *P* value threshold to 0.005 appears to be an editorial procedure worth pursuing, given its minimal costs and inconvenience to editors and reviewers. In a recent discussion of *P* values, Greenland [64] argued that, as with tobacco smoking, education, not prohibition, might be the best way to limit their misuse and its attendant harms. However, while outright prohibition might have proved as problematic with tobacco as it did with alcohol, there is little doubt that policies that have restricted the circumstances and places in which one can smoke (e.g., smoking bans in workplaces, restaurants and bars, public transport, places of entertainment, aircraft) made significant contributes to declining rates. Such policy changes restrict opportunities to smoke and decrease its social acceptability [65]. Our model suggests changing the *P* value threshold restricts the opportunity for researchers to find a “significant” (and publishable) result and, if the effort to produce such a result through data dredging becomes more arduous and extreme, its acceptability will, hopefully, decline over time. Researchers are more likely to see the absurd and unethical nature of trying to squeeze a *P*<0.005 result out of a data set as the analyses required become increasingly distant from those originally intended. This, over time, might help change the current research culture of many disciplines in which *P*-hacking is so easy it virtually goes unnoticed. As with smoking, a comprehensive approach to the problem of *P* values is required; we believe changing the threshold for statistical significance should be part of this approach.

Although the results of the simulations suggest there are benefits in terms of a reduction in published false positive results to be derived from changing the *P* value threshold from 0.05 to 0.005, there are also potential negative consequences that must be considered. First, there are compelling arguments to the effect that it is not the threshold used to designate “statistically significance” that is the problem with *P* values, but rather the very use of this statistic is problematic and should be discontinued [66]. Changing the *P* value threshold will therefore simply encourage the continued use of a statistical practice that should be abandoned altogether. Second, and more broadly, Greenland [67] contends that null hypothesis significance testing reinforces cognitive biases that are detrimental to the practice of science, specifically “dichotomania” (the tendency to misperceive quantities as dichotomous even when this is incorrect and misleading) and “nullism” (the assumption that false positives are more problematic than false negatives). From this perspective changing the threshold simply moves the point at which the dichotomy is made and does so in a manner that assumes an over-abundance of false positives is a problem that requires addressing (based, as noted in the introduction, on the observation that null findings are relatively rare in the published literature of the academic disciplines upon which our modeling assumptions are based).

In response, while we are sympathetic to both arguments and believe they have merit, it seems very unlikely that the many disciplines in which *P* values are widely used (and misused) will abandon them anytime soon. To paraphrase Goodman [68], *P* values are in the statistical air of a great many academic disciplines and, as such, even those statisticians who would prefer a Bayesian atmosphere must live and breathe them to survive. In addition, eradicating *P* values would require an alternative, with effect size estimates (and confidence intervals) and Bayesian models the most frequently suggested [66,69,70]. The few cases in which journals have required authors to report effect estimates and not *P* values have met with limited success, with researchers largely ignoring confidence intervals when presenting their results and continuing to focus on their “significance” [71]. As Finch et al. [72] observe, proper presentation and interpretation of effect sizes and confidence intervals would require prior upstream training in these methods for researchers in many disciplines and not just editorial stricture. There would be even more additional training required if the alternative to *P* values was Bayesian analysis. A recent survey found that almost half of 323 clinical trial medical researchers reported insufficient knowledge as the main reason they did not use Bayesian statistics [73]. Thus, changing the *P* value threshold seems, at this point, a modest proposal that might help correct the problem of false positive results identified in many social and behavioral academic disciplines.

A third potential problem with introducing a stricter threshold for statistical significance was found in the optimality models presented by Campbell and Gustafson [74], which show that reducing false positives in the published literature can lead to a depletion in the number of truly “breakthrough discoveries” appearing in academic journals. This applies also to our models as they require investigators expending more effort to publish fewer papers but with more reliable results. The extent to which journal editors want to balance publication of truly reliable and valid results against publishing truly novel and surprising results might depend on the subject matter of the discipline and the question being addressed in the research. In cases where a very pressing issue with substantial societal implications is being addressed, then less stringent requirements for “statistical significance” (i.e., *P*<0.05) might be warranted. In many cases, however, “breakthrough discoveries” will be of interest primarily to other academics in a particular field of research and requiring these to meet a more stringent standard of statistical significance before publication will likely not result in any major cost to society. It might also help reduce the so-called “decline effect” [75] whereby an initially positive (but false) research finding concerning a phenomenon fails to be replicated in subsequent studies but becomes resistant to falsification by virtual of its perceived novelty and early influence on the field. The persistence of non-reproduced work can, in fact, be quite large [76,77].

Sir Ronald Fisher, considered by many to have popularized “tests of significance” using *P* values, stated, with respect to the *P*<0.05 criterion, that “[a] scientific fact should be regarded as experimentally established only if a properly designed experiment *rarely fails* to give this level of significance” [78, p.85, Fisher’s emphasis]. That is to say, *P*<0.05 was intended as a screening tool, after which multiple replicates would be required for a “real” finding. Robinson and Wainer [79, p.264] emphasize that Fisher “understood science as a continuous and continuing process and viewed [what has come to be known as] ‘null hypothesis significance testing’ in that context.” Until the scientific research community can converge on longer-term and more challenging-to-implement interventions, a more onerous screening level may reduce the number of false positive publications as suggested by our models.
